# From bacteria to fish: ecotoxicological insights into sulfamethoxazole and trimethoprim

**DOI:** 10.1007/s11356-024-34659-y

**Published:** 2024-08-14

**Authors:** Bárbara S. Diogo, Sara Rodrigues, Oksana Golovko, Sara C. Antunes

**Affiliations:** 1 ICBAS, Instituto de Ciências Biomédicas de Abel Salazar, Universidade do Porto, Rua de Jorge Viterbo Ferreira, 228, 4050-313 Porto, Portugal; 2 CIMAR/CIIMAR, Centro Interdisciplinar de Investigação Marinha e Ambiental, Universidade do Porto, Terminal de Cruzeiros do Porto de Leixões, Avenida General Norton de Matos, S/N, 4450-208 Matosinhos, Portugal; 3 FCUP, Departamento de Biologia, Faculdade de Ciências, Universidade do Porto, Rua do Campo Alegre S/N, 4169-007 Porto, Portugal; 4 Department of Aquatic Sciences and Assessment, Swedish University of Agricultural Sciences (SLU), 75007 Uppsala, Sweden

**Keywords:** Antibiotics, Screening toxicity, Aquatic organisms, Bioassays, Biomarkers

## Abstract

Sulfamethoxazole (SMX) and trimethoprim (TRIM) are two of the most used antibiotics in the last 50 years, to prevent and treat bacterial infections; however, the available literature about toxicity to non-target organisms is quite discrepant and incomplete. This study aims to assess the SMX and TRIM ecotoxicological effects in standard species: *Aliivibrio fischeri* (bioluminescence inhibition), *Escherichia coli* ATCC 25922 (growth inhibition), *Lemna minor* (growth inhibition and biochemical biomarkers), *Daphnia magna* (immobilization/mortality, life history traits, and biochemical biomarkers), and *Danio rerio* (survival, hatching, abnormalities, and biochemical biomarkers). The species tested showed different acute sensitivities to SMX (*A. fischeri* < *D. magna* < *E. coli* < *L. minor*) and TRIM (*L. minor* < *A. fischeri* < *D. magna* < *E. coli*)*.* Overall, TRIM reveals less toxicity than SMX, except for *E. coli* (Ecotoxicological approach based on Antimicrobial Susceptibility Testing – EcoAST procedure). Both antibiotics affect individually (e.g., growth and survival) and sub-individually (e.g., antioxidant defenses) *L. minor*, *D. magna*, and *D. rerio*. This study allowed us to generate relevant data and fill gaps in the literature regarding the effects of SMX and TRIM in aquatic organisms. The here-obtained results can be used to (i) complete and re-evaluate the Safety Data Sheet to improve the assessment of environmental safety and management of national and international entities; (ii) clarify the environmental risks of these antibiotics in aquatic ecosystems reinforcing the inclusion in the 4th Watch List of priority substances to be monitored in whole inland waters by the Water Framework Directive; and (iii) combat the development of antimicrobial resistance, as well as supporting the definition of environmental measurements in the context of European One Health Action Plan. However, it is essential to continue studying these antibiotics to better understand their toxicity at ecologically relevant concentrations and their long-term effects under different climatic change scenarios.

**Supplementary Information:**

The online version contains supplementary material available at 10.1007/s11356-024-34659-y.

## Introduction

Sulfonamides (SAs) are a group of antibiotics widely used since the 1970s (Yousef et al. 2018), and due to its broad spectrum of action and low cost, it represents one of the oldest groups of antibiotics used (Park and Choi 2008). Although the consumption of SAs has increased slightly between 2011 and 2021 (from 0.54 to 0.58 Defined Daily Dose (DDD) per 1000 inhabitants per day) in the European Union (EU) (ECDC 2021), the consumption of these antibiotics, combined with diaminopyrimidines, increased from 0.46 to 0.53 DDD per 1000 inhabitants per day (ECDC 2021), between 2016 and 2021. Most formulations with SAs are produced in combination with synthetic diaminopyrimidines, since they act synergistically on specific targets in bacterial DNA synthesis (Daeseleire et al. 2017). One of the most consumed and produced combinations contains a 5:1 ratio of sulfamethoxazole (sulfonamide) and trimethoprim (diaminopyrimidine), respectively. Versporten et al. (2021) reported that this mixture (called cotrimoxazole and applied in human medicine for systemic treatments) represented the only antibiotic of the SAs group consumed in 6 EU countries in 2017 (Belgium, Bulgaria, Croatia, Hungary, Italy, and Luxembourg), since it provides an effective synergistic treatment against a broad spectrum of bacterial infections (e.g., infections of the urinary, respiratory and gastrointestinal tract) (Smilack 1999).

Sulfamethoxazole (SMX) is a bacteriostatic antibiotic, that represents one of the most prescribed and consumed SAs in Europe (Oliveira et al. 2019; Smilack 1999). SMX acts during an intermediate step of the formation of tetrahydrofolic acid, being a structural analog of para-aminobenzoic acid and competing with it to inhibit the synthesis of dihydrofolic acid (Brain et al. 2008). Trimethoprim (TRIM), also a bacteriostatic antibiotic, is responsible for inhibiting the enzyme dihydrofolate reductase, preventing the formation of the active metabolite of tetrahydrofolic acid (Masters et al. 2003). Together SMX and TRIM act sequentially to inhibit enzyme systems involved in the bacterial synthesis of tetrahydrofolic acid, affecting DNA synthesis (Masters et al. 2003). Thus, SMX and TRIM were two of the most used antibiotics of the last 50 years in human and veterinary medicine, aquaculture, and agriculture to prevent and treat the most diverse bacterial infections (e.g., urinary infections) (Carvalho and Santos 2016).

In the last few years, several authors have reported the detection of SMX and TRIM, at levels from ng/L to µg/L, in different environmental matrices worldwide (e.g., wastewater, freshwater, marine, and groundwater) (Carvalho and Santos 2016; Duan et al. 2022; Kovalakova et al. 2020). Carvalho and Santos (2016) considered that this increase is mainly caused by the direct discharge of wastewater treatment plants (WWTPs), where the sewage treatments may be ineffective, leakage of manure storage tanks, or leaching from farmland fertilized with manure. Due to the biological activity of antibiotics and their continuous entry into aquatic ecosystems, an increase in concentrations has been reported, with significant effects on non-target organisms at different trophic levels (e.g., bacteria, algae, microcrustaceans, and fish) (Diogo et al. 2023b). The antibiotics can induce adverse effects even at low concentrations, compromising different metabolic and physiological pathways (Rodrigues et al. 2016).

Although there is already some information on the potential toxicity of the mixture of the antibiotics (namely SMX and TRIM), the available data about their single effects, namely in the safety data sheet (SDS), is incomplete. Most SDS do not have disposable data, especially concerning acute and chronic toxicity (Sigma Aldrich 2022a, 2022b). Regarding the literature, different studies report the toxicity of SMX and TRIM to several aquatic organisms; however, these data are quite discrepant, namely in median effective concentration (EC_50_) (Duan et al. 2022). For the microcrustracean *Daphnia magna*, EC_50_ values vary between 43.97 and 189.2 mg/L to SMX (Drzymała and Kalka 2020; Kim et al. 2007) and between 92 and 167.2 mg/L to TRIM after 48 h of exposure (Kim et al. 2007; Park and Choi 2008). The same discrepancy was observed for the bacterium *Aliivibrio fischeri* after 30 min of exposure to SMX (EC_50_ between 0.05 and 100 mg/L) (Grinten et al. 2010; Osorio et al. 2016) and for the macrophyte *Lemna minor* after 7 days of TRIM exposure (EC_50_ between 27.43 and 215 mg/L) (BMG Engineering Ltd. 2011; De Liguoro et al. 2012).

Due to the wide use (human and veterinary medicine), concentrations detected in the environment, and possible toxicity to aquatic organisms, SMX and TRIM have been included in the 3rd and carried over to the 4th Watch List of priority substances to be monitored in inland waters by the Water Framework Directive (Cortes et al. 2020, 2022). These lists attempt to collect high-quality data to determine the risk that various substances could pose to aquatic ecosystems and human health (Niegowska et al. 2021).

The present study aims to fill in the knowledge gaps about the ecotoxicological effects of the antibiotics SMX and TRIM, in standard aquatic organisms. Thus, short-term assays were carried out with *Aliivibrio fischeri*, *Escherichia coli* (ATCC 25922), *Lemna minor*, *Daphnia magna*, and *Danio rerio*. Additionally, sub-individual parameters were used to evaluate the effect of antibiotics on different metabolic pathways and physiological functions in *L. minor* (total chlorophyll and carotenoids, catalase (CAT) and Glutathione S-transferases (GSTs) activities, malondialdehyde (MDA), and proline contents), in *D. magna* (CAT and GSTs activities, levels of thiobarbituric acid reactive substances (TBARS), and acetylcholinesterase (AChE) activity), and in *D. rerio* (CAT and GSTs activities, TBARS levels, and AChE activity).

## Material and methods

### Chemicals and test solutions

SMX and TRIM were acquired from Sigma-Aldrich (Table 1). The properties of antibiotics, stock solutions, and concentrations tested for the different organisms studied are listed in Table 1. Stock solutions were prepared by dilution of SMX and TRIM in the respective culture medium, which is specific for each species tested, and the range of concentrations tested was chosen based on literature data concerning their acute toxicity (e.g., Carvalho and Santos 2016; Drzymała and Kalka 2020; Duan et al. 2022).

**Table 1:** Properties of antibiotics sulfamethoxazole and trimethoprim, stock solutions (mg/L), range of concentrations tested (mg/L), and decay (%) of each antibiotic after 0-, 24-, 48-, 72-, 96-, and 168-h exposure

		Sulfamethoxazole (SMX)		Trimethoprim (TRIM)	
Characteristics	CAS n°	723–46-6		738–70-5	
	IUPAC name	4-Amino-N-(5-methyl-3-isoxazolyl)benzenesulfonamide, N1-(5-Methylisoxazol-3-yl)sulfanilamide		2,4-Diamino-5-(3,4,5-trimethoxybenzyl)pyrimidine	
	Molecular formula	C_10_H_11_N_3_O_3_S		C_14_H_18_N_4_O_3_	
	Molecular weight (g/mol)	253.28		290.3	
	Purity (%)	≥ 98.0		≥ 98.5	
Stock solutions and concentrations tested (mg/L)	*A. fischeri*	Stock solution: 100 16.8 to 100 (Factor dilution 1.25x)		Stock solution: 400 61.3 to 384.6 (Factor dilution 1.3x)	
	*E. coli*	Stock solution: 300 0.147 to 150 (Factor dilution 2x)		Stock solution: 400 0.0015 to 200 (Factor dilution 2x)	
	*L. minor*	Stock solution: 10 0.2 to 5 (Factor dilution 1.5x)		Stock solution: 450 147.1 to 450 (Factor dilution 1.15x)	
	*D. magna*	Stock solution: 200 Acute: 46.5 to 200 (Factor dilution 1.2x)		Stock solution: 200 Acute: 93.3 to 200 (Factor dilution 1.1x)	
		Stock solution: 78 Sub-chronic: 2.8 to 45 (Factor dilution 2x)		Stock solution: 50 Sub-chronic: 3.1 to 50 (Factor dilution 2x)	
	*D. rerio*	Stock solution: 2.5 0.156 to 2.5 (Factor dilution 2x)		Stock solution: 400 25 to 400 (Factor dilution 2x)	
		Analytical concentration (mg/L)	Decay (%)	Analytical concentration (mg/L)	Decay (%)
0 h		360	-	330	-
24 h		320	11.1	327	1.0
48 h		270	25.0	290	12.1
72 h		240	33.3	250	24.2
96 h		200	44.4	245	25.8
168 h		190	47.2	233	29.3

### Test organisms, maintenance, and bioassays conditions

A set of model organisms belonging to different trophic levels were selected to perform the present study, namely bacteria *Aliivibrio fischeri* (NRRL strain number B-11177) and *Escherichia coli* (ATCC 25922), the macrophyte *Lemna minor*, the cladoceran *Daphnia magna*, and the fish *Danio rerio*.

#### *Aliivibrio fischeri *assay

*Aliivibrio fischeri*, also known as *Vibrio fischeri*, is a marine bioluminescent bacterium used to assess the acute toxicity of compounds through Standard Microtox® assays. This assay was performed using the liquid samples procedure, according to standardized protocols (Microbics 1992), and started after the *A. fischeri* rehydration with a reconstitution solution. Bioluminescence evaluation was performed 30 min after exposure, at 9 concentrations of each antibiotic (Table 1), using the Microtox Model 500 toxicity system with an automatic luminescence recording, following the protocol of the Microtox® Acute Toxicity Basic Test Procedures manual–Modern Water. EC_50_ values and corresponding confidence intervals were used to report the toxicity values of each antibiotic.

#### *Escherichia coli* (ATCC 25922) assays

*Escherichia coli* is a Gram-negative bacterium, used as an indicator of water quality in several studies (e.g., Diogo et al. 2023c). The growth inhibition of *Escherichia coli ATCC® 25922™* after exposure to different concentrations of SMX and TRIM was evaluated according to the adaptations of EUCAST methodology for an ecotoxicological approach based on Antimicrobial Susceptibility Testing – EcoAST assay (for more details, *see* Supplementary material). Considering the maximum water solubility limit of the antibiotics understudy, in the EcoAST approach, the maximum possible concentrations tested were 150 mg/L of SMX and 200 mg/L of TRIM.

*E. coli* ATCC 25922 was initially grown in Muller Hinton agar medium and transferred into Muller Hinton liquid medium. Overnight, liquid cultures of *E. coli* were used to prepare a pre-inoculum of 5.0 × 10^5^ colony-forming units (CFU)/mL. The assays were performed in 96-well plates, and 4 replicates of each concentration were used (Table 1). Then, 100 µL of the Muller Hinton liquid medium and 100 µL of stock solutions of SMX or TRIM (300 and 400 mg/L, respectively, Table 1) were placed in a well. After that, each antibiotic was serially diluted (Table 1), and 100 µL of the *E. coli* inoculum was added (10^5^ CFU/mL) to each well. Media control (200 µL of Muller Hinton liquid medium), antibiotics control (100 µL of stock solution of antibiotic (SMX or TRIM) + 100 µL of Muller Hinton liquid medium), and growth control (100 µL of Muller Hinton liquid medium + 100 µL of *E. coli* inoculum) were made. The microplates were incubated at 37 °C for 24 h in light absence, and after the exposure period, the bacterial growth was measured spectrophotometrically at a wavelength of 600 nm. The results were expressed in percentage of inhibition growth and were used to obtain EC_50_ and EC_80_ (24 h) and corresponding confidence intervals.

An additional assay was performed to determine the minimum bactericidal concentration (MBC), defined as the lowest concentration that causes complete death of *E. coli* (≥ 99.9%). For this assay, three drops of 10 µL from specific concentrations of the EcoAST procedure (the concentration below the MIC and for all concentrations above the MIC) were inoculated in Muller Hinton agar medium and incubated at 37 °C. Growth and, consequently, cell viability were assessed after 24 h, and the absence of bacterial growth was used as an indicator to define the MBC value (CLSI 1999).

#### *Lemna minor* assays

Growth inhibition assays of *Lemna minor* were conducted in general accordance to standard guidelines (OECD 2006). The assays were performed in glass vials, with four replicates per treatment, containing 100 mL of each antibiotic concentration (Table 1) and 9 fronds of *L. minor*. A negative control with 100 mL Steinberg medium was also performed. The glass vials were maintained under continuous light (24 h, ~ 7000 lx) and at constant temperature (23 ± 1 °C). The results were expressed in yield (7 days) and used to obtain EC_50_ and corresponding confidence intervals (OECD 2006). At the end of the assay, the fronds were washed with distilled water, dried with blotting paper, and weighed. The fronds were then stored in Eppendorf microtubes at − 80 °C for posterior quantification of total chlorophyll and carotenoid contents and determination of specific biochemical endpoints (CAT and GSTs activities, and MDA and proline contents). All the biochemical biomarkers were performed according to Pinto et al. (2021) and Diogo et al. (2023c). Since at the highest SMX concentration, the growth (and consequently the available biomass) was reduced, it was not possible to evaluate all parameters and it was decided not to measure the proline content.

#### *Daphniamagna* assays

*Daphnia magna* acute immobilization assay (OECD No. 202) was performed in general accordance with the standard guideline (OECD 2004), for acute immobilization test (48 h), with some adaptations. The neonates were exposed to SMX and TRIM concentrations (Table 1) and negative control (ASTM hard water; ASTM 2023). For this assay, 8 replicates of each treatment were prepared in glass vessels, containing 30 mL of antibiotics concentrations, and 5 organisms (neonates with < 24 h old and born between the 3rd and the 5th broods). The glass vessels were maintained under controlled conditions of temperature (20 ± 1 °C) and photoperiod (16 h^L^:8 h^D^; ~ 5000 lx). After 24 h and 48 h of exposure, the number of organisms normal/mobile (N), with irregular swimming behavior (ISB), immobile (I), and dead (D), was observed according to the different categorizations defined in Diogo et al. (2023a). After the exposure period (48 h), three pools (with 6 or 7 organisms) from each antibiotic concentration were prepared for posterior quantification of lipid peroxidation (thiobarbituric acid reactive substances (TBARS) levels) and neurotoxicity (acetylcholinesterase (AChE) activity) (Diogo et al. 2023a). Due to the higher mortality values verified in the highest concentrations tested, it was not possible to quantify TBARS levels and AChE activity in organisms exposed to 136.9, 166.7, and 200 mg/L of SMX and 181.8 and 200 mg/L of TRIM.

*D. magna* sub-chronic assays were performed following the guideline OECD No. 211 (OECD 2012) with the modifications described by Diogo et al. (2023a). For each treatment, 21 individualized replicates (neonates less than 24 h old and born between the 3rd and the 5th broods) were placed in glass vessels with 30 mL of ASTM (negative control) or antibiotic concentrations (Table 1). A range of SMX and TRIM concentrations (Table 1) was selected to guarantee that the inferior value of the confidence interval of acute EC_5_ was not exceeded (Table 1). The assay was conducted in the same conditions of temperature and light described in the acute immobilization test. The test medium was entirely renewed every 48 h, and the organisms were fed with the green microalgae *Raphidocelis subcapitata* (in a ratio of 3.0 × 10^5^ cells/mL/day). The mortality and life history parameters were observed daily, and neonates born during the assay were counted and discharged. After 10 days of antibiotic exposures, life-history endpoints were quantified: age at first reproduction, N1 fecundity, somatic growth rate, and rate of population increase (Masteling et al. 2016). Additionally, three pools (with 3 or 4 organisms) from each treatment were prepared to assess different biomarkers, namely oxidative stress (CAT and GSTs activities, and TBARS levels) and neurotoxicity (AChE activity) (Diogo et al. 2023a).

#### *Danio rerio* embryos assays

*Danio rerio* embryo acute toxicity assays were conducted according to the standard Fish Embryo Acute Toxicity (FET) Test (OECD No. 236; OECD 2013), with some modifications. The embryos used in the experiment were born from a laboratory broodstock (wildtype AB) of the zebrafish facility at CIIMAR—Interdisciplinary Centre of Marine and Environmental Research (Matosinhos, Portugal). After fertilization, and as quickly as possible, viable eggs (without irregularities) were selected, and the assays were started. The assays were performed in 24-well microplates, with 20 replicates per treatment (concentration) and one embryo per replicate. Each well contained 2 mL of each antibiotic concentration (Table 1) or sterile dechlorinated water (control group). The microplates were incubated under a controlled temperature (26 ± 1 °C) and photoperiod (16 h^L^:8 h^D^; ~ 1000 lx), for 96 h. The mortality was evaluated throughout the assay (after 24, 48, 72, and 96 h of exposure), and all the coagulated eggs or dead embryos were removed. For daily (24, 48, 72, and 96 h), all the morphological abnormalities observed were registered. The observations of embryos/larvae were performed using a binocular stereoscope LeicaMZ 75, with an attached camera Leica DFC 290. After 96 h of exposure, three pools (with 3 or 2 organisms) from each antibiotic concentration were prepared to assess oxidative stress (CAT and GSTs activities, and TBARS levels) and neurotoxicity (AChE activity) effects (Diogo et al. 2023a).

### Analytical quantification of SMX and TRIM

To evaluate the stability of SMX and TRIM, when exposed to temperature (20 ± 2 °C) and light (i.e., day/night), stock solutions of 400 mg/L of SMX and 400 mg/L of TRIM were prepared with distilled water. Then, the solutions were distributed in several falcons (50 mL of solution in each one) and stored in controlled conditions of temperature (20 ± 2 °C) and photoperiod (16 h^L^:8 h^D^; ~ 5000 lx) for different periods (24, 48, 72, 96, and 168 h). To determine analytical concentrations of antibiotics, samples from 0 h (immediately after preparing the stock solutions) and after 24, 48, 72, 96, and 168 h (7 days) were collected and frozen at − 20 °C. The selection of these periods for collecting samples, for chemical analysis, was defined based on the different exposure times for different standard bioassays. The % decay over time of the antibiotics was determined with reference to the analytical concentrations quantified at 0 h. For the analytical quantification, the samples were filtered using a regenerated cellulose syringe filter (0.22 mm pore). One milliliter of the filtered sample was spiked with 10 ng of internal standards of trimethoprim-13C-D3 and sulfamethoxazole-D4 per aliquot of sample. The samples were analyzed by a DIONEX UltiMate 3000 ultra-high pressure liquid chromatography (UPLC) system (Thermo Scientific, Waltham, MA, USA) coupled to a triple quadrupole mass spectrometer (MS/MS) (TSQ QUANTIVA, Thermo SCIENTIFIC, Waltham, MA, USA). An Acquity UPLC BEH-C18 column (Waters, 100 mm × 2.1 i.d., 1.7 µm particle size from Waters Corporation, Manchester, UK) was used. The injection volume was 10 µL for all samples. A heated electrospray ionization (H-ESI) was used to ionize the target compound. The spray voltage was set to static: positive ion (V) 3500. Nitrogen (purity > 99.999%) was used as a sheath gas (50 arbitrary units), auxiliary gas (15 arbitrary units), and sweep gas (2 arbitrary units). The vaporizer was heated to 400 °C and the capillary to 325 °C. The mobile phase consisted of Milli-Q with 5 mM ammonium acetate and acetonitrile. The flow rate was 0.5 mL/min and the run time was 15 min. Xcalibur software (Thermo Fisher Scientific, San Jose, CA, USA) was used to optimize the instrument methods and running of samples. The obtained data were evaluated using TraceFinderTM 3.3. software (Thermo Fisher). The linearity of the calibration curve was tested in the range from 0.1 to 1000 ng/mL. The limit of quantification (LOQs) was calculated as one-quarter of the lowest calibration point in the calibration curve where the relative standard deviation of the average response factor was < 30%. LOQ were 0.01 mg/L for TRIM and 0.012 mg/L for SMX. The precision of the method was evaluated by the repeatability of the study. For this purpose, all samples were prepared in triplicates. No studied compounds were detected in blank samples.

### Statistical analysis

EC_50_ values, and respective 95% CIs (using the delta method), were determined by fitting a nonlinear concentration–response toxicity model (LL3) to the *L. minor* yield data and the *A. fischeri* inhibition of bioluminescence data using the drc package (Ritz and Streibig 2005) for R software. All the EC_50_ values were calculated based on nominal concentrations. The yield and inhibition of bioluminescence were modeled as a continuous variable using a three-parameter logistic model, where the lower asymptotes of the curve were fixed to 0, following Ritz (2010). The estimation of LC_50_ values for *D. magna* was based on the number of dead organisms, while EC_5_ and EC_50_ values were based on the sum of the results of the effects categorization of the dead (D), immobile (I), and irregular swimming behavior (ISB) binomial data (using the R package “drc”; (Ritz and Streibig 2005)), with a special case of the log-logistic dose–response model, where the asymptotes of the curve are fixed to be 1 (all organisms are dead, immobilized or with irregular swimming behavior) and 0 (none are immobile), following the rationale of Ritz (2010). According to the classification proposed by EU-Directive 93/677/ECC (EC 1996), the chemicals can be classified as very toxic (EC_50_ < 1 mg/L), toxic (1 < EC_50_ < 10 mg/L), harmful (10 < EC_50_ < 100 mg/L), and non-toxic (EC_50_ > 100 mg/L) for aquatic species.

Bioassay results and physiological endpoints were tested for normality by the Shapiro–Wilk test and homogeneity of variances by the Levene test. A one-way ANOVA was conducted for all biomarkers results, followed by the Dunnett test (which was carried out to determine differences between the antibiotic concentrations and the control group). All the statistical analysis was done using SPSS Statistics v26, using 0.05 as the level of significance.

## Results and discussion

### Analytical quantification of SMX and TRIM

The analytical concentrations and percentage of decay of SMX and TRIM quantified after 0, 24, 48, 72, 96, and 168 h (7 days) are shown in Table 1. A greater decay of SMX (11.1%) was verified in the first 24 h compared to TRIM (1.0%). After 48 h, both antibiotics increased the decay rate (25.0% of SMX and 12.1% of TRIM); however, with the number of hours increasing, a higher decay of SMX (47.2% after 168 h) was observed compared to TRIM (29.3% after 168 h).

### *Aliivibrio fischeri*

The results obtained in the *A. fischeri* bioluminescence assay (EC_50_ values and the respective 95% confidence intervals) after exposure to SMX and TRIM are shown in Table 2. According to the EU-Directive 93/677/ECC (EC 1996), SMX was considered harmful for *A. fischeri* (EC_50_ = 69.9 mg/L), while TRIM was considered non-toxic (EC_50_ = 230 mg/L) (Table 2). The literature data are quite discrepant regarding the sensitivity of *A. fischeri* to SMX and TRIM. Osorio et al. (2016) recorded that SMX was non-toxic for *A. fischeri*, after 30 min of exposure, (EC_50_ = 100 mg/L), while Isidori et al. (2005) and Ferrari et al. (2004) considered SMX harmful (EC_50_ = 23.3 and 84 mg/L, respectively). SMX was also considered harmful to *A. fischeri* by Grabarczyk et al. (2020) who reported an EC_50_ = 51.8 mg/L, which is in the same range value obtained in the present study (Table 2). Grinten et al. (2010) studied the effects of antibiotics understudy on *A. fischeri* and revealed that this bacterium was less sensitive to SMX (EC_50_ (30 min) > 1.5 mg/L) and TRIM (EC_50_ (30 min) > 0.28 mg/L) than bacterial plates specifically sensitive to sulfonamides. Although studies with TRIM are scarce, this antibiotic had already been considered non-toxic for *A. fischeri* after 5 and 15 min of exposure (EC_50_ = 165.1 mg/L and 176.7 mg/L, respectively) by Kim et al. (2007).

**Table 2:** E(L)C_50_ values and CI (confidence intervals 95%) for *Aliivibrio fischeri*, *Escherichia coli* ATCC 25922, *Lemna minor*, and *Daphnia magna* after exposure to sulfamethoxazole (SMX) and trimethoprim (TRIM). Values of EC_80_ for *E. coli* and EC_5_ for *D. magna* are also presented

	Sulfamethoxazole (mg/L)	Trimethoprim (mg/L)
*Aliivibrio fischeri* (30 min)	**EC**_**50**_** = 69.9** (34.0–106)	**EC**_**50**_** = 230** (< 0–512)
*Escherichia coli* (24 h)	**EC**_**50**_** = 17.1** (15.3–18.9)	**EC**_**50**_** = 0.31** (0.27–0.34)
	**EC**_**80**_** = 20.4** (18.1–22.8)	**EC**_**80**_** = 0.49** (0.40–0.58)
*Lemna minor* (7 d)	**EC**_**50**_** = 2.11** (1.65–2.58)	**EC**_**50**_** = 305** (261–350)
*Daphnia magna* (48 h)	**EC**_**5**_** = 49.4** (45.0–53.9)	**EC**_**5**_** = 81.3** (75.3–87.2)
	**EC**_**50**_** = 68.8** (65.7–71.8)	**EC**_**50**_** = 98.6** (95.7–101)
	**LC**_**50**_** = 124** (117–131)	**LC**_**50**_** = 168** (162–173)

### *Escherichia coli* ATCC 25922

EC_50_ and EC_80_ values (and the respective confidence intervals) of both antibiotics for *E. coli* are present in Table 2. SMX was classified as toxic (EC_50_ = 17.1 mg/L) and TRIM (EC_50_ = 0.31 mg/L) was very toxic for *E. coli*, according to EC (1996). Although EUCAST databases do not contain the values of MIC of SMX for this strain, Trujillo-Casarreal et al. (2023) reported that the growth of the same strain of *E. coli* can be reduced by 80% when exposed to 32 mg/L of SMX. According to the results obtained, 20.4 mg/L of SMX reduced 80% of *E. coli* growth (Table 2). Regarding TRIM, the literature reported that concentrations between 0.5 and 2 mg/L of TRIM can cause an inhibition of *E. coli* growth of 80% (EUCAST 2023). In fact, our results corroborated this since, for TRIM an EC_80_ = 0.49 mg/L was determined (Table 2). Aagaard et al. (1991) showed that in standardized conditions (in Muller-Hinton medium and pH 7), 0.3 mg/L of TRIM can reduce the growth of *E. coli* by 90%, which demonstrates the resistance capability of this species to TRIM, since in the present study, in the same conditions (medium and pH), 0.39 mg/L of TRIM only reduced the growth by 67.44%.

**Fig. 1:**
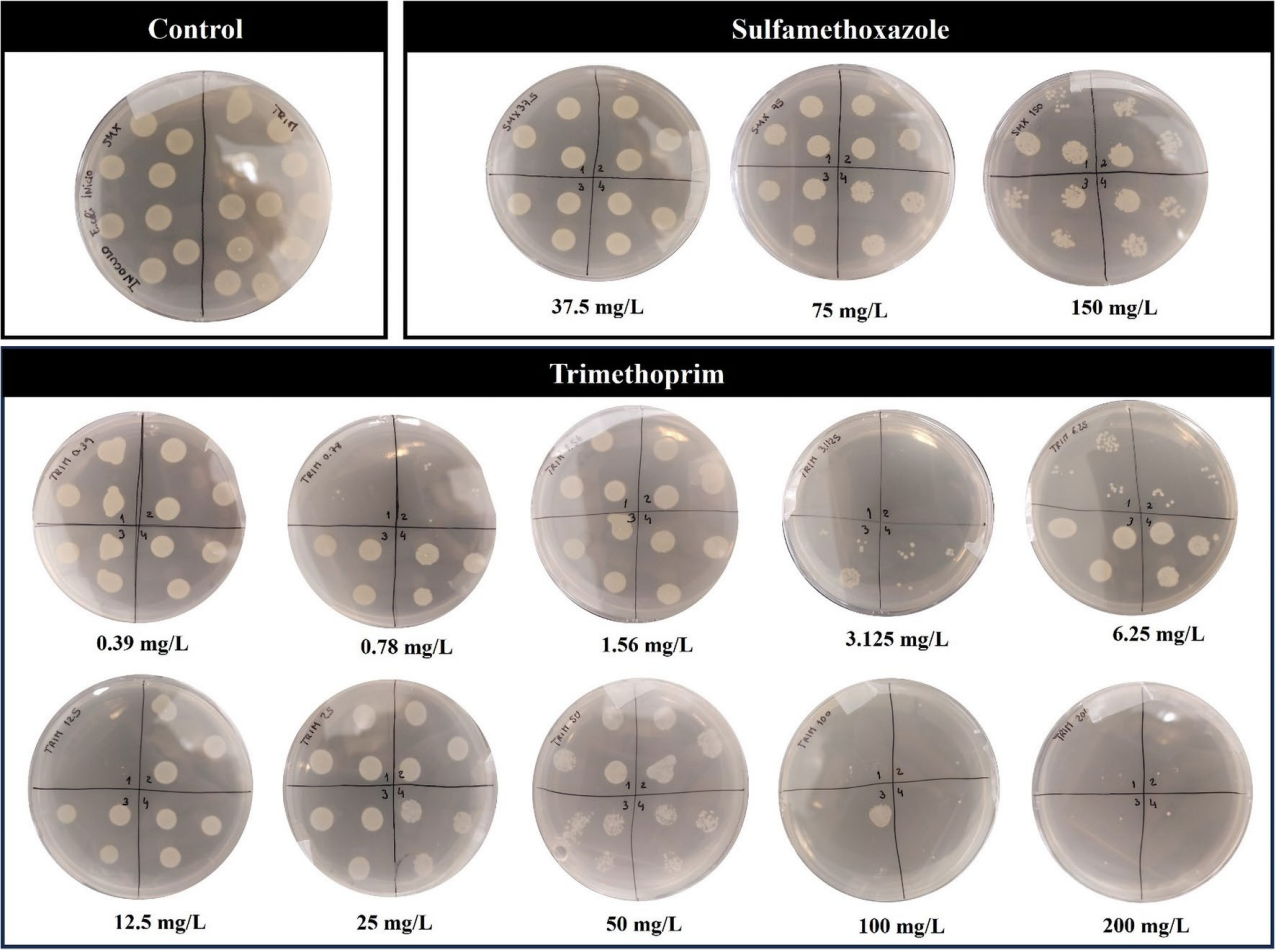
Growth of *Escherichia coli* ATCC 25922, in control conditions, and after exposure to a range of sulfamethoxazole and trimethoprim concentrations (mg/L)

Figure 1 shows the growth levels of *E. coli* ATCC 25922 after exposure to SMX and TRIM concentrations. It was shown that the higher concentrations of both antibiotics affect drastically the growth and cell viability of *E. coli* (Fig. 1). The literature considers SMX and TRIM bacteriostatic antibiotics (antibiotics that inhibit the growth and reproduction of bacteria without provoking death) and non-bactericidal antibiotics (antibiotics without the ability to cause death or bacteria destruction) (Bernatová et al. 2013). However, the present study demonstrated that although the tested concentrations of SMX were not bactericidal (is bacteriostatic since the highest concentrations tested—37.5, 75, and 150 mg/L—did not cause growth inhibition > 99.9%; Fig. 1), the highest concentrations of TRIM (100 and 200 mg/L) caused the death of the strain under study (Fig. 1) showed a minimum bactericidal concentration (MBC) > 100 mg/L. Based on the literature, both antibiotics inhibit the folate pathway in bacteria; however, TRIM interferes at a later stage (compared to SMX), demonstrating bacteriostatic properties (Masters et al. 2003). Furthermore, TRIM alone can be a potentially bactericidal agent for microorganisms (Burman 1986).

### *Lemna minor*

*Lemna minor* growth inhibition results (EC_50_ values and the respective confidence intervals) are shown in Table 2. According to the EU-Directive 93/677/ECC, the antibiotics SMX and TRIM showed to be toxic (EC_50_ = 2.11 mg/L) and non-toxic (EC_50_ = 305 mg/L) to *L. minor*, respectively, (EC 1996). Several authors reported the phytotoxicity of different antibiotics (Brain et al. 2004), namely of the group sulfonamides (e.g., sulfamethoxazole, sulfadimethoxine, sulfamethazine, and sulfathione) (Białk-Bielińska et al. 2011). Grabarczyk et al. (2020) reported an EC_50_ of 3.07 mg/L for *L. minor* after exposure to 7 days of SMX (similar to our results). A similar EC_50_ value (3.67 mg/L of SMX) was also reported by Aubakirova et al. (2017). Brain et al. (2004) showed the high toxicity of SMX for *L. gibba* causing a reduction in wet weight and number of fronds, after exposure to concentrations lower than 1 mg/L. Cheong et al. (2020) showed that sulfonamide antibiotics cause folate deficiency, affecting cell division, growth, and development of plants. This group of antibiotics is a structural analog of *p*-aminobenzoic acid, which was already described as being able to inhibit the same pathway as in bacteria (synthesis of tetrahydrofolic acid) and plants, including aquatic plants, such as *Lemna* spp. (Białk-Bielińska et al. 2011; Brain et al. 2004). The presence of SMX can significantly impact aquatic plant communities, leading to a decrease in primary production, habitat loss, and reduced biodiversity.

Regarding the toxicity of TRIM for *L. minor*, the results obtained in this study (Table 2) corroborate with the few studies in the literature that report EC_50_ values greater than 100 mg/L (Straub 2013). De Liguoro et al. (2012) studied the effects of TRIM (6.25 to 100 mg/L) on the frond number and fresh weight of *L. minor* and reported that concentrations higher than 25 mg/L caused a significant decrease in frond number and fresh weight. TRIM also affects the folate synthesis pathway (Masters et al. 2003), and like other antifolic agents (e.g., sulfonamides) can affect the development of plants (the decrease of folate levels affects the DNA, RNA, and protein synthesis and consequently decrease the cell growth and development). Although SMX and TRIM act sequentially to inhibit enzyme systems involved in the folate pathway, these antibiotics affect the same species differently. Typically, SMX is more toxic since it can interfere with multiple cellular processes (Xu et al. 2022).

**Fig. 2:**
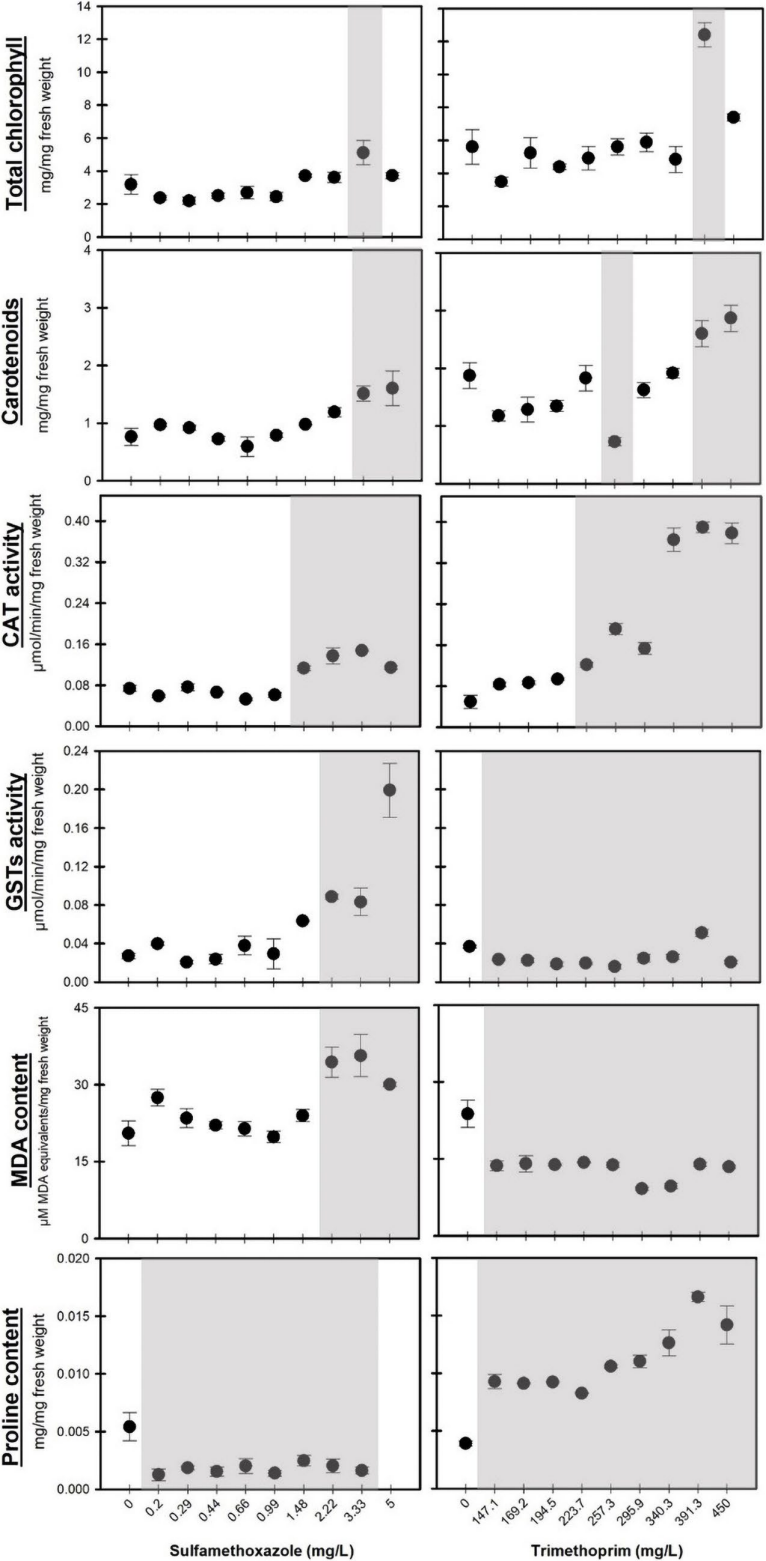
Results of physiological and biochemical biomarkers in *Lemna minor* after exposure (7 days) to a range of sulfamethoxazole (left) and trimethoprim (right) concentrations (mg/L). Data are expressed as mean ± standard error. Grey shadows represent significant differences compared to the control treatment (Dunnett’s test, *p* < 0.05). The lack of data for proline at the highest concentration of SMX is due to the absence of *L. minor* biomass

To recognize the biochemical effects that occurred in *L. minor* after acute exposure to SMX and TRIM, different physiological and biochemical biomarkers were determined (Fig. 2). The total content of chlorophyll in *L. minor* increased significantly (*F*_[9, 29]_ = 6.037, *p* < 0.001; *F*_[9, 29]_ = 5.889, *p* < 0.001, respectively) after exposure to 3.33 mg/L of SMX, while an increase in carotenoid content was observed in concentrations up to 3.33 mg/L (Fig. 2). Regarding TRIM exposure, total chlorophyll content significantly increased after exposure to 391.3 mg/L (*F*_[9, 29]_ = 13.993, *p* < 0.001), whereas carotenoids content showed a non-monotonic response, with a decrease after exposure to 257.3 mg/L and an increase after exposure to concentrations up to 391.3 mg/L (*F*_[9, 29]_ = 14.218, *p* < 0.001). Although variations in the synthesis/degradation of pigments represent one of the typical signals of oxidative stress in plants, these parameters are not always the most sensitive (Diogo et al. 2023c). In this study, significant variations in the pigment content of *L. minor* after exposure to both antibiotics were recorded; however, no response pattern occurred (Fig. 2).

Zhang et al. (2021) have already reported that a significant increase and decrease in photosynthetic pigments may result from a defense mechanism of the organism against stress. Furthermore, it was reported that the increase in chlorophyll content may arise as a defense mechanism to eliminate reactive oxygen species (ROS) in photosynthetic cells (Zhang et al. 2021). According to Sharma et al. (2020), most antibiotics cause a decrease in the pigment content in plants; however, this response may vary with the type of antibiotic, concentrations, and exposure time. Zhang et al. (2021) also found that chlorophyll and carotenoid contents of microalgae *Chlorella vulgaris* vary with the SMX concentrations (mg/L) and time of exposure (days). Dong et al. (2020) studied the effects of SMX on *Microcystis aeruginosa* and *C. vulgaris* pigment contents and reported that in low concentrations (20–50 mg/L of SMX), minimal effects were observed in chlorophyll *a* concentration, while at higher concentrations (80–200 mg/L), a significant decrease was observed.

Figure 2 shows the results obtained in CAT and GSTs activities and MDA content in *L. minor* after exposure 7 days to different concentrations of SMX and TRIM. A significant increase in CAT and GSTs activities (*F*_[9, 29]_ = 30.682, *p* < 0.001; *F*_[9, 29]_ = 21.609, *p* < 0.001, respectively) and MDA content (*F*_[9, 29]_ = 7.702, *p* < 0.001) was observed with the highest concentrations of SMX tested (≥ 2.22 mg/L). CAT represents one of the first enzymes involved in the antioxidant defense system (responsible for the catalysis of hydrogen peroxide in water) (Diogo et al. 2023c), while GSTs represent a group of enzymes that constitute an important component of the phase II detoxification system (responsible for conjugating GSH with electrophilic compounds, making their excretion easier) (Diogo et al. 2023a). Thus, the results demonstrate that although the detoxification and antioxidant defense pathways were mobilized (significant increase in CAT and GSTs activities, Fig. 2), these enzymes were not able to neutralize and prevent lipid peroxidation in *L. minor*. The increase in MDA content indicates that SMX affected *L. minor* causing membrane damage (lipid peroxidation) and potentially affecting the fitness of this macrophyte. Zhang et al. (2021) reported that despite the increase in antioxidant defense enzymes (such as SOD, CAT, and glutathione peroxidase (GPx)), concentrations higher than 0.5 and 0.015 mg/L of SMX induce lipid peroxidation in *Synechococcus* sp. and *Raphidocelis subcapitata*, respectively. Despite the limited number of studies on the toxic effects of SMX in *L. minor*, several authors reported that this antibiotic caused lipid peroxidation in other aquatic species, namely microalgae and fish (Lin et al. 2014; Zhang et al. 2021). Regarding proline content (Fig. 2), a significant decrease was observed after exposure to all SMX concentrations tested (*F*_[8, 26]_ = 8.797, *p* < 0.001). It has already been demonstrated that proline content can be used as a sensitive biomarker of oxidative stress, since it is an amino acid that plays an important role in plant physiology, namely in cellular homeostasis and redox balance (e.g., Nunes et al. 2014; Diogo et al. 2023c). A decrease in proline content, as observed after exposure of *L. minor* to SMX (Fig. 2), may also reinforce the possibility of oxidative-based effects (Nunes et al. 2014). In this case, proline rather than being an intermediary in regulatory processes may have acted as a direct antioxidant (scavenging free radicals), as discussed by other authors, namely Bohnert and Jensen (1996) and Diogo et al. (2023c).

Effects on oxidative stress and detoxification pathways promoted by TRIM are still unknown, and studies carried out with this antibiotic and macrophytes are scarce. In this study, after *L. minor* exposure to TRIM, a significant increase in CAT activity (*F*_[9, 29]_ = 118.494, *p* < 0.001) was observed at the highest concentrations tested (> 223.7 mg/L, Fig. 2). A significant decrease was observed in GSTs activity (*F*_[9, 29]_ = 17.516, *p* < 0.001) after exposure to almost all concentrations tested (except with 391.3 mg/L of TRIM, where a significant increase was observed, Fig. 2). The decrease in GSTs activity may be associated with lower bioavailability of reduced glutathione (GSH) for the conjugation process mediated by GSTs, since GSH can be mobilized as a non-enzymatic antioxidant defense (Rodrigues et al. 2023). The MDA content also decreased significantly (*F*_[9, 29]_ = 14.248, *p* < 0.001) after exposure to all concentrations tested. Thus, the results obtained demonstrate that, after TRIM exposure, the antioxidant defense system (CAT and GSTs activities) of *L. minor* was enough to prevent lipid peroxidation. The results of the present study showed that TRIM can induce the activation of the antioxidant defense of *L. minor*, as reported by Gomes et al. (2020) for other antibiotics (e.g., amoxicillin, oxytetracycline, and enrofloxacin).

A significant increase in proline content was observed after exposure to all the concentrations tested of TRIM (*F*_[9, 29]_ = 25.032, *p* < 0.001; Fig. 2). The most common response to oxidative stress in plants involves an increase in proline content (Nunes et al. 2014), as observed after exposure *L. minor* to TRIM. These results reinforce the ability of *L. minor* to neutralize the damage caused by this antibiotic, avoiding a complete scenario of oxidative stress. Indeed, other studies already reported that after exposure to different antibiotics, namely norfloxacin (Zhao et al. 2022); carbenicillin and cefotaxime (Qin et al. 2011); oxytetracycline, streptomycin, and sulfadimethoxine (a sulfonamide antibiotic) (Tasho et al. 2020); the variation in proline content in plants acted as ROS scavenger and contributed to osmotic adjustments; stabilization and protection of the integrity of membranes (Tasho et al. 2020).

### *Daphnia magna*

#### Immobilization assays

Table 2 shows the results of EC_5_, EC_50_, and LC_50_ of *D. magna* after exposure to different concentrations of SMX and TRIM. Acute immobilization assay proved that both antibiotics were considered harmful to *D. magna* (Table 2); however, SMX was revealed to be more toxic than TRIM (EC 1996). Several authors reported *D. magna* EC_50_ of SMX with some discrepancy, from 43.97 (Drzymała and Kalka 2020) to 189.2 mg/L (Kim et al. 2007). In our study, the EC_50_ value obtained (EC_50_ = 68.8 mg/L SMX; Table 2) was similar to the value obtained by Osorio et al. (2016) (EC_50_ = 75.3 mg/L SMX). In contrast, the only LC_50_ value found in the literature for *D. magna* (LC_50_ (48 h) = 234.18 mg/L of SMX; (Lu et al. 2013)) is higher than that observed in the present study (124 mg/L). Despite the scarcity of studies with TRIM, the results obtained in this study corroborate with the toxicity values recorded in the literature, namely an EC_50_ (48 h) in the order of 100 mg/L (Kim et al. 2007; Park and Choi 2008) and an LC_50_ (48 h) of 167.4 mg/L (Kim et al. 2007).

**Fig. 3:**
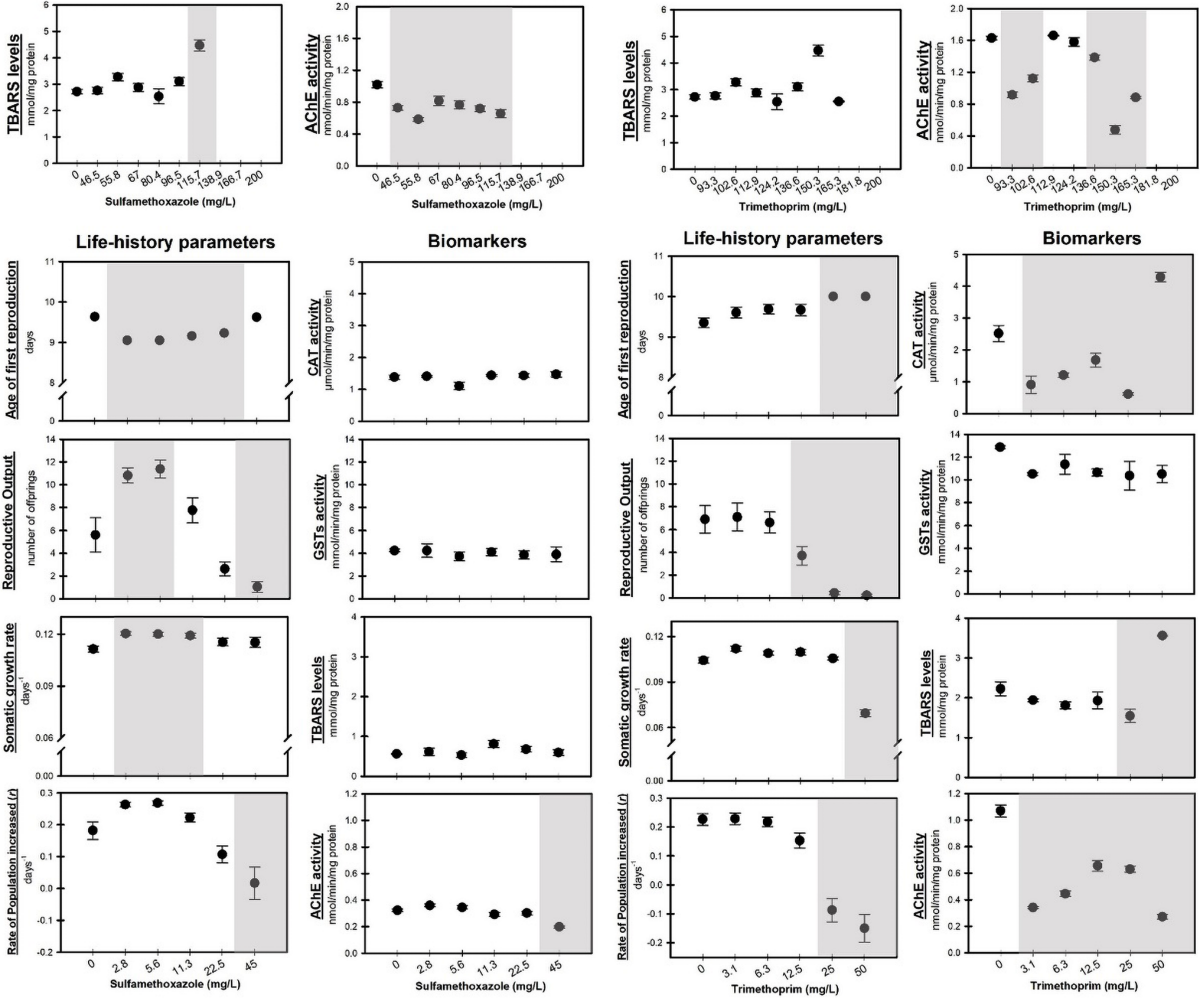
Results of biochemical biomarkers (TBARS levels and AChE activity) in *Daphnia magna* after acute exposure (48 h) to sulfamethoxazole (top, left) and trimethoprim (top, right). Life-history parameters and biochemical biomarkers (CAT and GSTs activities, TBARS levels, and AChE activity) after *D. magna* sub-chronic exposure (10 days) to sulfamethoxazole (below, left) and trimethoprim (below, right). Data are expressed as mean ± standard error. Grey shadows represent significant differences from the control treatment (Dunnett’s test, *p* < 0.05). The lack of data of acute biochemical biomarkers at the highest concentrations of SMX and TRIM is due to the absence of *D. magna* biomass

At the end of the acute exposure, a biochemical analysis was conducted (Fig. 3). A significant increase in TBARS levels (*F*_[6, 20]_ = 13.743, *p* < 0.001) was observed after exposure to 115.7 mg/L of SMX, revealing that this antibiotic can cause lipid peroxidation in *D. magna*. On the other hand, no significant changes were registered in TBARS levels after 48-h exposure to TRIM (*F*_[7, 23]_ = 1.119, *p* = 0.398), which reinforces the higher toxicity of SMX for *D. magna*. Kim et al. (2009) studied the acute effect of 9.5, 30, 94.9, and 300 mg/L of sulfathiazole (a sulfonamide antibiotic) on *D. magna* and reported that the oxidative stress caused by the highest concentrations was responsible for the high mortality observed. Studies reporting the acute biochemical effects of TRIM on *D. magna* are limited; however, several authors have already reported that this antibiotic can cause an increase in ROS levels and induce oxidative stress in other species (e.g., copepods and mussels) (Binelli et al. 2009; Han et al. 2016).

Regarding AChE activity, a significant decrease was recorded after acute exposure to all concentrations of SMX (*F*_[6, 20]_ = 10.360, *p* < 0.001; Fig. 3) and at the lowest (93.3 and 102.6 mg/L) and highest TRIM concentrations tested (136.6 and 150.0 mg/L—showed a non-monotonic dose–response *F*_[7, 23]_ = 140.692, *p* < 0.001; Fig. 3). SMX and TRIM demonstrated their impact on swimming behavior, leading to irregular swimming and immobilization of *D. magna*, with the increase of antibiotics concentrations (effects recorded in acute assay). This indicates that SMX and TRIM can induce neurotoxic effects and alter the nervous system. There are several antibiotics (e.g., fluoroquinolone class) that have indirect effects on the nervous system or enzymatic activity of *D. magna*, namely inhibiting AChE activity and causing neurotoxic effects (Dionísio et al. 2020). Several authors reported that SMX and TRIM can indirectly interfere with the synthesis of tetrahydrobiopterin (BH4), which is a cofactor in several enzymatic reactions, including those involved in the biosynthesis of neurotransmitters (e.g., serotonin and dopamine) (Eichwald et al. 2023; Haruki et al. 2013). Haruki et al. (2013) showed that SMX can inhibit sepiapterin reductase, an enzyme that catalyzes the final step of BH4 biosynthesis, and thereby significantly decreases the levels of neurotransmitters. On the other hand, TRIM causes the inhibition of dihydrofolate reductase (DHFR)—an enzyme responsible for the reduction of dihydrobiopterin to BH4—and consequently causes a decrease in BH4 (Parashar et al. 2016). The deficient levels of BH4 can cause ROS increase, neurotransmitter dysfunctions, and movement and neurological disorders in different organisms (Eichwald et al. 2023). Despite the biological function of BH4 in invertebrates is limited, several authors reported that this enzyme plays an important role in the physiological and immune processes of phylogenetically different species, that have biochemical mechanisms and metabolic pathways similar to those of *D. magna* (Wang et al. 2021; Zhu et al. 2022).

#### Sub-chronic assays

Sub-chronic assays revealed that *D. magna* life-history parameters were significantly affected by SMX and TRIM after 10 days of exposure (Fig. 3). SMX caused significant anticipation in the age of first reproduction (AFR; *F*_[5, 90]_ = 6.782, *p* < 0.001) at all concentrations except the highest and an increase in reproductive output (RO; *F*_[5, 125]_ = 21.081, *p* < 0.001) and somatic growth rate (SGR; *F*_[5, 118]_ = 3.805, *p* = 0.003) at the lowest concentrations. A significant drop in the rate of population increase (*r*; *F*_[5, 125]_ = 13.567, *p* < 0.001) was detected at 45.0 mg/L SMX. Lu et al. (2013) observed that exposure of *D. magna* to 3.33 and 10 mg/L SMX caused a significant delay of AFR and a decrease in fecundity after chronic exposure (21 days). Other sulfonamides reveal different effects on the reproductive capacity of *D. magna* (chronic assays) (Park and Choi 2008)*.*

Regarding TRIM sub-chronic results, a significant delay in AFR was observed at 25.0 and 50 mg/L (*F*_[5, 71]_ = 2.939, *p* < 0.019), while a significant decrease was observed in RO after exposure ≥ 12.5 mg/L (*F*_[5, 125]_ = 31.590, *p* < 0.001). Additionally, a significant decrease was observed in SGR (*F*_[7, 111]_ = 86.411, *p* < 0.001) and *r* (*F*_[5, 125]_ = 31.590, *p* < 0.001), at the highest TRIM concentrations tested (Fig. 3). Park and Choi (2008) observed a delay of AFR in *D. magna* after exposure to 20 mg/L TRIM. In addition, they also observed that higher TRIM concentrations affected the population growth of two cladocerans species (*D. magna* and *M. macrocopa*). The here-obtained results corroborate the findings of De Liguoro et al. (2012) which reported that ≥ 12.5 mg/L TRIM significantly decreases the number of neonates per female after 21 days of exposure. Dalla Bona et al. (2015) also reported the inhibition effects of TRIM (13, 25, and 50 mg/L) on the reproduction and growth of *D. magna* after chronic exposure.

The results of biochemical biomarkers after sub-chronic exposure in *D. magna* are presented in Fig. 3. No significant alterations were observed in CAT (*F*_[5, 17]_ = 3.584, *p* = 0.033) and GSTs (*F*_[5, 17]_ = 0.765, *p* = 0.592) activities and TBARS levels (*F*_[5, 17]_ = 2.164, *p* = 0.127), after sub-chronic exposure to SMX. These results reveal that the antioxidant defense was not affected, and lipid peroxidation was not observed in *D. magna* after 10 days of exposure to SMX. The same pattern (no significant changes in CAT and GSTs activities) was observed after exposure of the mussel *Mytilus galloprovincialis* to 0.5, 50, and 500 µg/L SMX for 6 days (Chen et al. 2021). Serra-Compte et al. (2019) also concluded that glutathione reductase (GRed), GPx, CAT, and GSTs activities did not undergo significant changes, and that lipid peroxidation was not observed after 96 h of exposure to 10 µg/L of SMX in *M. galloprovincialis*. Tokanová et al. (2021) studied the sub-chronic effect of SMX (50, 100, and 500 µg/L for 14 days) in zebrafish and showed that, despite no significant changes in oxidative stress biomarkers (GRed, GPx, and GSTs activities), the lipid peroxidation increased with the increase of SMX concentrations.

AChE activity of *D. magna* was affected by the antibiotics under investigation, after sub-chronic exposure (Fig. 3). It decreased significantly at the highest concentration of SMX (45 mg/L; *F*_[5, 17]_ = 26.364, *p* < 0.001; Fig. 3). The same trend was observed in *Carassius auratus* after 7 days of exposure to 50 mg/L SMX (Li et al. 2012). Moreover, Zhang et al. (2023) reported a significant decrease in AChE activity after sub-chronic exposure (14 days) at 100 and 1000 µg/L SMX, evidencing a disruption in the locomotor behavior of *D. magna*, due to neurological injury.

Regarding TRIM biochemical results, no significant alterations were observed in GSTs activities (*F*_[5, 17]_ = 1.797, *p* = 0.188); however, a significant decrease in CAT activity (*F*_[5, 17]_ = 51.987, *p* < 0.001) was observed, except at the highest concentration tested (50 mg/L), where a significant increase was observed. TBARS levels decreased significantly at 25 mg/L TRIM (*F*_[5, 17]_ = 27.721, *p* < 0.001); however, the opposite was observed at the highest concentration (50 mg/L). This reveals that probably the antioxidant defenses were enough to prevent lipid peroxidation at lower TRIM concentrations but failed at the highest concentration tested. Similar results were observed in other studies performed with TRIM, which reported that to maintain the ROS balance and prevent oxidative damage, different antioxidant enzymes were activated (e.g., SOD, CAT, GRed) (Binelli et al. 2009; Fernandez et al. 2022). Fernandez et al. (2022) studied the chronic effects (21 days) of TRIM in *Sparus aurata* and verified that oxidative damage did not occur when CAT and GSTs activities increased significantly. Significant alterations in SOD, CAT, and GSTs activities were also observed in *Dreissena polymorpha* (freshwater mussel) after exposure to TRIM (0.29, 0.87, and 2.9 µg/L), for 96 h (Binelli et al. 2009). Likewise, when copepod *Tigriopus japonicus* was exposed to 100 mg/L TRIM for 96 h, a scenario of oxidative stress, affecting its growth and reproduction, was observed (Han et al. 2016).

*D. magna* AChE activity decreased significantly (*F*_[5, 17]_ = 100.470, p < 0.001; Fig. 3) after exposure to all TRIM concentrations tested. The mode of action of SMX and TRIM is not directly related to neurotransmission; however, as mentioned previously (*see* *Daphnia magna* immobilization results) these antibiotics can affect the BH4 pathway and consequently cause neurotoxic effects in different model organisms (Eichwald et al. 2023). Furthermore, by interfering with the folic acid and carbonic anhydrase (CAs) synthesis pathways, SMX and TRIM are capable of disturbing neurotransmitters and causing endocrine alterations, affecting development and neurobehavior in different organisms (Huo et al. 2023).

### *Danio rerio* embryos

**Fig. 4:**
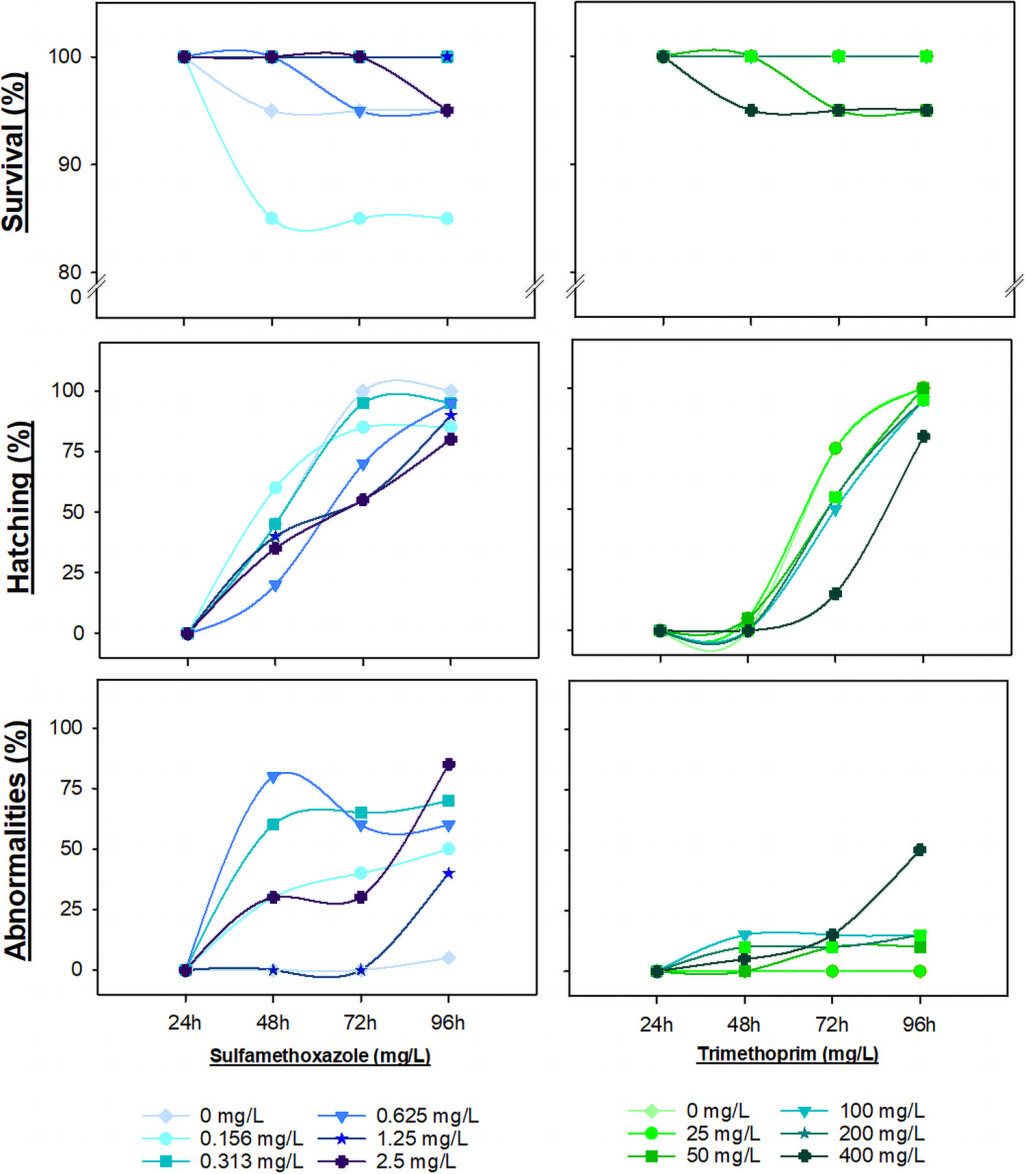
Percentage (%) of survival, hatch rate, and abnormalities of *Danio rerio* embryos after 24-, 48-, 72-, and 96-h exposure to a range of sulfamethoxazole (left) and trimethoprim (right) concentrations (mg/L)

The percentage of survival, hatching, and abnormalities observed in *D. rerio* embryos after 24, 48, 72, and 96 h of exposure to SMX and TRIM is presented in Fig. 4. The survival rate of zebrafish embryos was affected after exposure ≥ 0.156 mg/L SMX, the lowest percentage of survival (85%) being recorded after 48 h (Fig. 4). The lowest percentage of survival (95%) was recorded for TRIM after 48-h exposure to 400 mg/L (Fig. 4). At the end of the exposure period (96 h), a decrease in hatching was observed when the embryos were exposed to all the concentrations of SMX and the highest concentrations of TRIM (100, 200, and 400 mg/L) (Fig. 4). Previous studies already showed that 5 mg/L SMX causes mortality in 30 to 40% of the zebrafish embryos, after 96 h of exposure, while 1 mg/L of SMX causes a decrease of 11.5% in hatching (Iftikhar et al. 2023; Lin et al. 2013). Regarding TRIM exposure, one study reported no sublethal effects (e.g., malformations) in zebrafish embryos, after exposure to 10 mg/L (for 24, 48, and 144 h post-fertilization) (Carlsson et al. 2013). Richards and Cole (2006) tested a range of TRIM concentrations (1 to 100 mg/L) in *Xenopus laevis* embryos and did not report malformations or mortality, after 96 h of exposure.

**Fig. 5:**
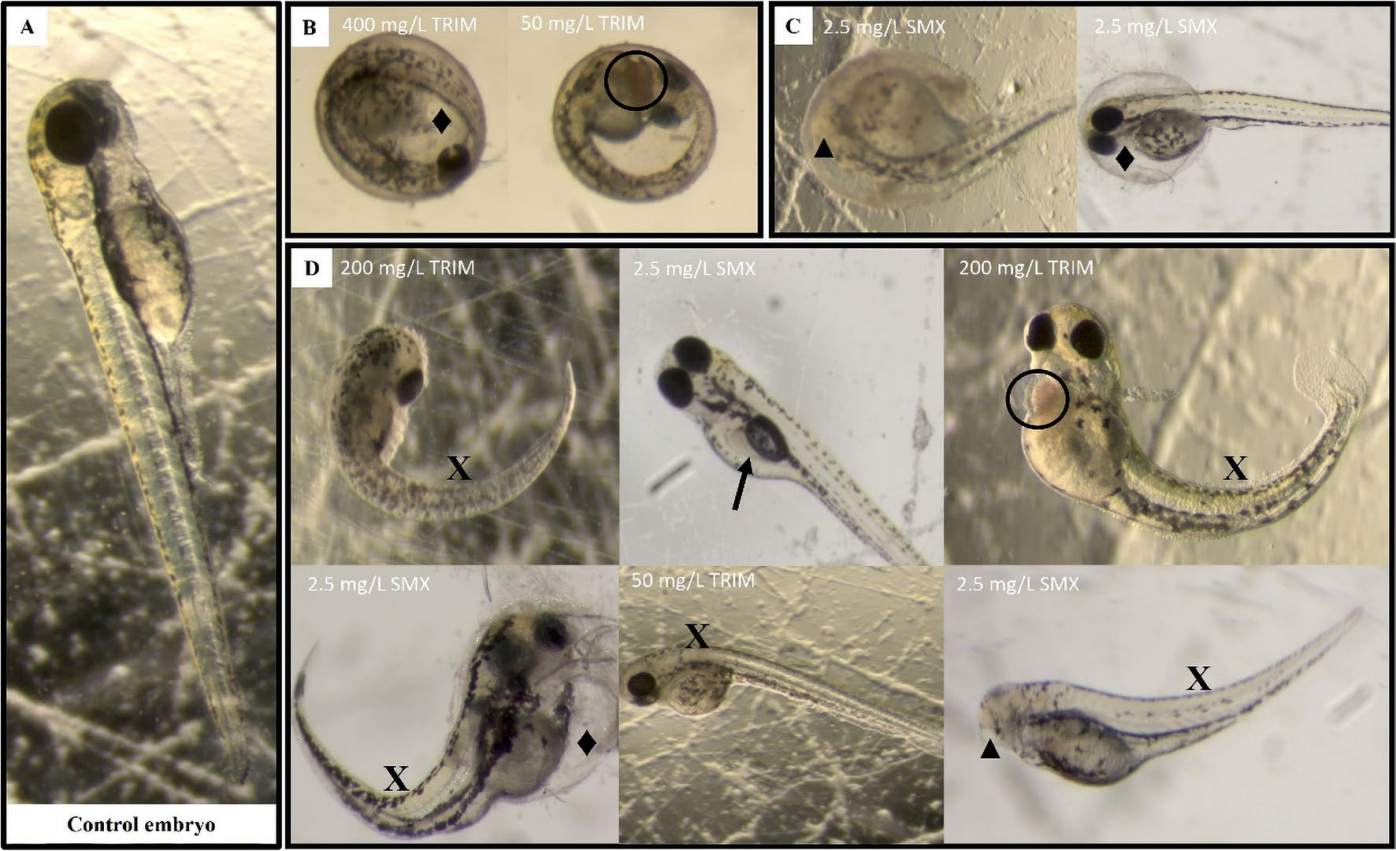
Examples of abnormalities observed in *Danio rerio* embryos after 96-h exposure to a range of sulfamethoxazole (SMX) and trimethoprim (TRIM) concentrations (mg/L). **A** Control embryo after 96 h of exposure. **B** Unhatched, **C** partially hatched, and **D** hatched embryos. Black diamond suit (**♦)**, pericardial and yolk sac edemas; white circle (**○**), hemagglutination; black up-pointing triangle (**▲**), head and eyes malformations; rightwards arrow (** →**), swim bladder enlarged; Latin capital letter X (**X**), spinal curvatures

Several abnormalities and the respective percentages observed for each concentration of SMX and TRIM, after 96 h of exposure, are presented in Table 3. Regarding the control group, only 5% of the control embryos showed some morphological alterations, namely swim bladder enlarged, which is considered insignificant according to OECD (2013). The most common abnormalities observed after antibiotic exposure were swim bladder enlarged (all SMX concentrations and highest TRIM concentration), body curvature (all SMX concentrations and ≥ 50 mg/L of TRIM), and pericardial edema with 15% and 5% of embryos after 2.5 mg/L of SMX and ≥ 100 mg/L of TRIM, respectively (Table 3; Fig. 5). Other abnormalities were occasionally observed (Table 3; Fig. 5), namely head and eye malformations (Fig. 5C and D), hemagglutination (Fig. 5D), and yolk sac edemas (Fig. 5B, C and D). After 96-h exposure, both SMX and TRIM caused morphological malformations at all concentrations tested, reaching 85% and 50% at the highest, i.e., 2.5 and 400 mg/L, respectively. However, despite TRIM causing fewer abnormalities (≤ 50%), an increase in the abnormality percentage was observed, across the tested TRIM concentrations (Fig. 4).

**Table 3:** Percentage (%) of abnormalities observed on *Danio rerio* after 96-h exposure to a range of sulfamethoxazole and trimethoprim (mg/L) concentrations

		Sulfamethoxazole (mg/L)						Trimethoprim (mg/L)					
		0	0.156	0.313	0.625	1.25	2.5	0	25	50	100	200	400
Abnormalities (%)	Pericardial edema	0	0	5	0	0	15	0	0	0	5	5	5
	Yolk sac edema	0	5	0	0	0	0	0	0	0	0	0	0
	Hemagglutination	0	0	0	0	0	0	0	0	5	0	5	0
	Head malformations	0	0	5	0	0	5	0	0	0	0	10	0
	Eyes malformations	0	0	0	0	0	5	0	0	0	5	5	0
	Swim bladder enlarged	5	30	30	50	35	40	0	0	0	0	0	35
	Body curvature	0	15	30	10	5	20	0	0	5	5	5	10

The abnormalities identified in the present study (Table 3; Fig. 5) have already been described by Huo et al. (2023). These authors assessed the effects of sulfonamides (e.g., SMX, sulfadiazine, sulfisoxazole), in the different stages of development of zebrafish (24, 48, and 96 h post-fertilization) and recorded swim bladder with defects, tail deformations, pericardial edemas, and body axial deformities, which are non-specific alterations (may arise due to exposure to different compounds). Although it is more common for several compounds to lead to swim bladder loss or inflation in zebrafish, in the here-present study, the embryos exposed to all concentrations of SMX and to the highest concentration of TRIM showed an accelerated swim bladder development (Fig. 5D, 2.5 mg/L SMX). Qiang et al. (2016) have already demonstrated that this phenomenon can happen when zebrafish are exposed to other types of compounds (e.g., carbamazepine, an anticonvulsant). Although there are no studies concerning antibiotics that show mechanistic justification for this phenomenon, Gao and Yang (2023) highlight that the Wnt pathway (which regulates crucial aspects of cell fate determination, cell migration, cell polarity, neural patterning, and organogenesis during embryonic development) is very important during the development of the swim bladder of fish embryos. Due to the high conservation of the Wnt pathway across species (Winata et al. 2009; Yin et al. 2011), many chemicals, such as antibiotics, could likely interfere with this pathway in zebrafish embryos and consequently affect early swim bladder development. Yin et al. (2011) reported that some compounds (e.g., flame retardants and metals) can inhibit the Wnt and Hedgehog signaling pathways, disrupting the organization of precursor cells in the three layers of the swim bladder, and causing abnormal bladder development in zebrafish embryos.

Lin et al. (2013) detected different malformations, namely yolk sac edemas, hemagglutination, axial malformation, and different levels of tail bending in zebrafish, after exposure to concentrations lower than 1 mg/L of SMX, as described in the here-present results (Table 3, Figs. 4 and 5). Iftikhar et al. (2023) showed that the abnormalities and developmental effects observed in zebrafish after exposure to SMX may be explained by the mechanism of action of sulfonamides, since these antibiotics can affect the pre-epiboly cells and inhibit embryonic growth. Furthermore, sulfonamides, when causing epibolic dysfunction, can impair the development of the body axis and induce axial malformations. The appearance of yolk sac edema in zebrafish embryos was also reported as a consequence of the loss of epiboly action caused by sulfonamides (Iftikhar et al. 2023).

Another mechanism that may affect the development and central nervous system of the zebrafish embryo is the thyroid hormonal system, which regulates neuronal signaling in the early embryonic stages of zebrafish (Aderemi et al. 2020). Sulfonamides are considered thyroglobulin (Tg) deregulators, causing dilation and degranulation of the rough endoplasmic reticulum, which can result in a decrease in Tg secretion levels and a consequent hypothyroidism (Aderemi et al. 2020; Iftikhar et al. 2023; Lin et al. 2013). Lin et al. (2013) suggested that the tail-bending deformities observed in embryos exposed to sulfonamides are a consequence of the altered development of the nervous system, affected by hypothyroidism. In addition, in their study, it was verified that some of the embryos were unable to break the chorion and were not able to fully hatch after 72 h of exposure to SMX (Lin et al. 2013), a fact also recorded in the here-presented study. This hatching delay observed after SMX and TRIM exposure (Figs. 4 and 5) can be explained by the chemical mode of action of these antibiotics since by affecting folate metabolism, SMX and TRIM affect DNA, RNA, and protein synthesis and consequently decrease cell division (Iftikhar et al. 2023; Lin et al. 2013).

**Fig. 6:**
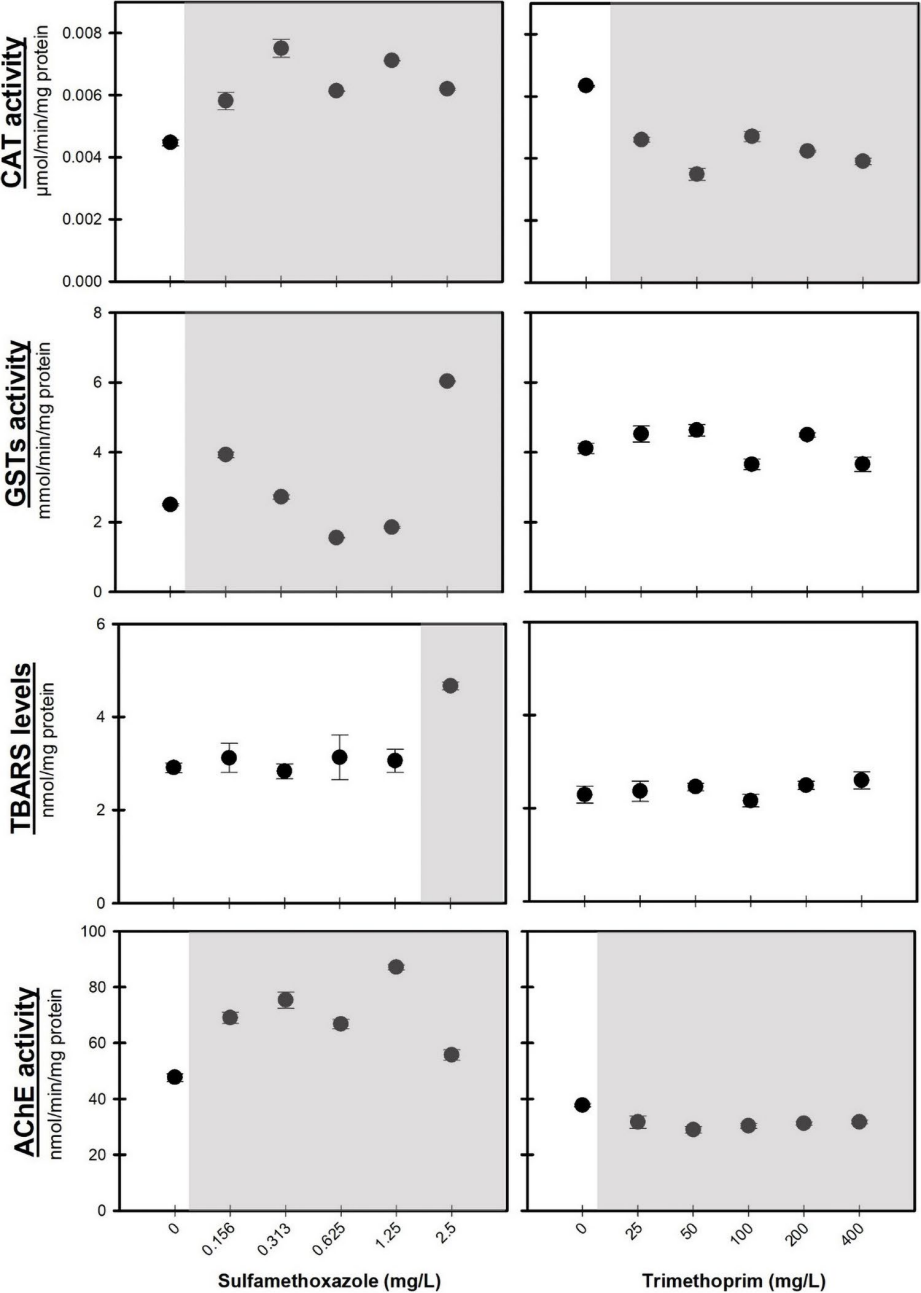
Results of biochemical biomarkers (CAT and GSTs activities, TBARS levels, and AChE activity) in embryos of *Danio rerio* after acute exposure (96 h) to a range of sulfamethoxazole (left) and trimethoprim (right) concentrations (mg/L). Grey shadows represent significant differences from the control treatment (Dunnett’s test, *p* < 0.05)

The results of biochemical biomarkers (CAT and GSTs activities, TBARS levels, and AChE activity), after acute exposure of *D. rerio* embryos to SMX and TRIM, are shown in Fig. 6. A significant increase was observed in CAT activity (*F*_[5, 17]_ = 39.657, *p* < 0.001) after exposure to all concentrations of SMX. Similar results were observed in GSTs activity after exposure to 0.156, 0.313, and 2.5 mg/L, while a significant decrease was observed at 0.625 and 1.25 mg/L of SMX (*F*_[5, 17]_ = 1236.313, *p* < 0.001). Only after exposure to the highest concentration of SMX (2.5 mg/L) was detected a significant increase in TBARS levels (*F*_[5, 17]_ = 6.537, *p* < 0.001). These results may indicate that antioxidant defense enzymes efficiently prevent lipid peroxidation at low SMX concentrations; however, their activity was insufficient at the highest tested concentration (2.5 mg/L) (Fig. 6). As reported by Yan et al. (2016) after exposure to SMX, the increased enzymatic activities (e.g., CAT) of zebrafish reflected the occurrence of oxidative stress and appeared to try to convert the ROS into harmless metabolites. Similar to the here-obtained results, Lin et al. (2014) already showed that SMX induces or inhibits the activity of CAT in zebrafish (after 7 days post-fertilization), depending on the concentration tested and the exposure time. Furthermore, the study also reported that the sulfonamide degradation can result in different metabolites with oxidation abilities, which can lead to the lipid peroxidation of membranes, consequently increasing TBARS levels (as observed in the present study and corroborated by their findings). Yang et al. (2020) reported that concentrations < 2 mg/L of another sulfonamide (sulfamethazine) induced physiological changes during the embryonic stages of zebrafish (e.g., heart rate). Furthermore, the same authors also demonstrated that 0.0002, 0.02, and 2 mg/L of sulfamethazine can cause redox imbalance in *D. rerio* embryos, increasing SOD activity and TBARS levels.

In terms of AChE activity, a significant increase was observed after exposure to all concentrations of SMX (*F*_[5, 17]_ = 54.496, *p* < 0.001). Despite no studies reporting the effects of these antibiotics on zebrafish AChE activity, other authors have already reported neurotoxic effects of antibiotics in zebrafish (Lin et al. 2013; Yang et al. 2020). Lin et al. (2013) evaluated the effect of different antibiotics (e.g., SMX, sulfadiazine, and sulfadimidine) on the integrative neuronal function of zebrafish larvae through the study of spontaneous swimming activity. The authors reported that low concentrations (< 0.01 mg/L) of these antibiotics can cause neurotoxic effects (decreasing the zebrafish spontaneous movements and coordination). Zebrafish embryos exposed to environmental concentrations of sulfonamides (0.001 mg/L) also showed alterations of the nervous system, increased heartbeat rate, and decreased spontaneous swimming activity (Lin et al. 2014), which can compromise the normal development and behavior of this organism. Hormones and endocrine-disrupting chemicals were already described with the ability to exhibit a non-monotonic dose–response (Vandenberg et al. 2012; Rodrigues et al. 2019), and our results seem to present a similar response (Fig. 6, CAT, GSTs, and AChE activities), although it has never been reported for these antibiotics.

Regarding TRIM results, a significant decrease in CAT activity was observed (*F*_[5, 17]_ = 74.327, *p* < 0.001), while no significant changes were observed in GSTs activity (*F*_[5, 17]_ = 6.849, *p* < 0.003) and TBARS levels (*F*_[5, 17]_ = 1.000, *p* = 0.458). AChE activity decreased significantly after exposure to all concentrations of TRIM (*F*_[5, 17]_ = 6.847, *p* < 0.001). Although there is no information about the effects of TRIM on the activity of antioxidant enzymes in zebrafish embryos, other studies report disruptive and neurological effects of TRIM in other embryos. Villa et al. (2018) showed that 400 mg/L of TRIM causes a slowdown in behavioral activities (e.g., average speed and distance covered) of *Diamesa zernyi* larvae, after 96 h of exposure. Huo et al. (2023) consider that the decrease in folic acid synthesis caused by exposure to antifolate antibiotics (e.g., sulfonamides and trimethoprim) is responsible for causing behavioral and significant changes in gene expression in zebrafish (e.g., related to folic acid synthesis, carbonic anhydrase, and neurotransmitter pathways). Moreover, the inhibition of the expression of key enzyme genes (e.g., *pah*, *th*, *tph1 a*) in zebrafish neurotransmitter synthesis, after exposure to these antibiotics, affects the neurobehavior and development of fish (Huo et al. 2023). Even more, according to Dorman (2000), sulfonamides can be considered gamma-aminobutyric acid (GABA) agonists, causing acute neurological effects. Thereby, Huo et al. (2023) suggest that antibiotics that affect the folic acid synthesis (as SMX and TRIM) can affect the carbonic anhydrases (CAs) gene expression and consequently modulate the release of GABA, which represents a potential risk of neurotoxicity for zebrafish (the inhibition of CAs affect the choroid plexus, GABAergic activity, and the plasma membrane of neurons). Similar to what was observed in biochemical biomarker results after SMX exposure, TRIM exposure also exhibits a non-monotonic dose–response (Fig. 6).

## Conclusions

The present study provided relevant information about the toxicity of the antibiotics sulfamethoxazole and trimethoprim, for aquatic standard organisms. SMX was generally harmful or toxic, impacting several species and their physiological pathways, while TRIM was less toxic but still capable of causing significant biochemical, cellular, and individual-level effects.

Significant ecotoxicological data were obtained that provide information regarding the environmental impact of SMX and TRIM and can be used to complete and re-evaluate the Safety Data Sheet for a better assessment of the environmental safety and management of national and international entities. Moreover, these data allow us to classify the hazard posed by antibiotics (SMX and TRIM) in aquatic ecosystems and reinforce the inclusion of these compounds in the 4th Watch List of priority substances to be monitored in whole inland waters by the Water Framework Directive. These findings are also essential if we consider the strong commitment of European and national countries’ policies to combat the development of antimicrobial resistance, as well as the European One Health Action Plan. However, further investigations to understand the effects of these antibiotics on different metabolic pathways and physiological functions of aquatic organisms are crucial. Furthermore, it is essential to study the long-term effect of ecologically relevant concentrations of sulfamethoxazole and trimethoprim, as well as their mixture, on non-target organisms and under different climatic change scenarios.

## Supplementary Information

Below is the link to the electronic supplementary material.

Supplementary file1 (DOCX 45.1 KB)

## Data Availability

All the data are included in the manuscript.
