# Supplementary material for: From bacteria to fish: ecotoxicological insights into sulfamethoxazole and trimethoprim
Source: Environ Sci Pollut Res Int. 2024 Aug 14;31(39):52233–52. doi: 10.1007/s11356-024-34659-y (PMC11374860; doi:10.1007/s11356-024-34659-y)
Supplement: Supplementary file 1 — Supplementary file1 (DOCX 45.1 KB) [file 11356_2024_34659_MOESM1_ESM.docx]

**Proposal and assessment of the EUCAST methodology for an ecotoxicological approach**

EcoAST procedure: Ecotoxicological approach based on Antimicrobial Susceptibility Testing

According to the European Committee on Antimicrobial Susceptibility Testing (EUCAST) methodology (EUCAST 2022) the lowest concentration of antibiotics able to inhibit bacterial growth in 80 %, compared to the growth control (without antibiotic), is considered the minimum inhibitory concentration (MIC). MIC assays are mostly associated with antimicrobial sensitivity studies with applications in medicine, however, these assays can also be adapted to assess environmental risk and toxicity of chemical substances, which can reach ecosystems and affect non-target organisms. Nevertheless, the concentrations used in MIC assays are predefined and clinically relevant (between 0.002 and 512 mg/L, dilution factor 2). Considering the solubility of TRIM [400 mg/L, (Sigma Aldrich, 2022)], if the volumes proposed by the EUCAST methodology (see below) were respected, we would be limited and would not be able to test high concentrations (up to 32 mg/L). Therefore, considering the objective of the present study (evaluate the ecotoxicological risk of the antibiotics for *Escherichia coli* ATCC 25922), the volumes and the concentrations proposed by EUCAST methodology were adapted (see below), for an ecotoxicological approach (EcoAST procedure: Ecotoxicological approach based on Antimicrobial Susceptibility Testing). To assess the adaptations made and considering that the MIC value for TRIM is already defined by EUCAST (2022), an assay was carried out with this antibiotic respecting the EUCAST methodology, and another assay with a few adaptations (EcoAST procedure), simultaneously. At the end of the assays (EUCAST Methodology *vs* EcoAST procedure), the results were analyzed and compared to perceive whether the variations made to the EUCAST methodology caused deviations from the MIC concentration for TRIM already defined in the literature. Furthermore, with the EcoAST procedure, it is possible to reliably determine the concentration that causes 80% of effects (in this case, inhibition of bacterial growth), that is, the EC_80_.

**EUCAST methodology**

*E. coli* ATCC 25922 was initially grown in Muller Hinton agar medium and transferred into Muller Hinton liquid medium (both acquired from Sigma Aldrich). Overnight liquid cultures of *E. coli* were used. The assays were performed in 96-well plates, and 4 replicates of each concentration were made. According to the EUCAST methodology (EUCAST 2022), 25 µL of the liquid medium (Muller Hinton, pH 7) and 25 µL of stock solutions of TRIM (256 mg/L – prepared in Milli Q water) were added to each well of microplates. Then, the antibiotic was serially diluted (dilution factor 2x) to obtain the defined concentrations of EUCAST methodology (Table S1). Afterward, 175 µL of the *E. coli* inoculum was added (10^5^ CFU/mL) to each well. Media control (200 µL of Muller Hinton liquid medium), TRIM control (25 µL of stock solution of TRIM + 175 µL of Muller Hinton liquid medium), and growth control (25 µL of Muller Hinton liquid medium + 175 µL of *E.coli* inoculum) were made. Due to the water solubility limit of the compound under study (400 mg /L of TRIM; (Sigma Aldrich 2022)], the concentrations tested were presented in Table S1. The microplates were incubated at 37 ºC for 24 h in light absence. After the exposure period (24 h) the bacterial growth was measured spectrophotometrically at a wavelength of 600 nm (EUCAST 2022) to determine the MIC. The results were expressed in the percentage of inhibition of growth.

**EcoAST procedure**

To evaluate the effects of antibiotics in bacterial growth, using an ecotoxicological approach, some adaptations were made, essentially in terms of the concentrations tested (maximum tested in EUCAST methodology: 32 mg/L of TRIM; maximum tested in ecotoxicological approach: 200 mg/L of TRIM), volumes used (EUCAST methodology: 25 µL of stock solutions of antibiotics + 175 µL of *E.coli* inoculum; EcoAST: 100 µL of stock solutions of antibiotics + 100 µL of *E.coli* inoculum) and final results to consider. To assess the adaptations of the ecotoxicological approach, two assays were carried out using the antibiotic Trimethoprim (TRIM): one according to the EUCAST methodology and the other EcoAST procedure. In both methodologies (EUCAST *vs* EcoAST), the microplates were incubated at 37 ºC for 24 h in light absence conditions, and after the exposure period, the bacterial growth was measured spectrophotometrically at a wavelength of 600 nm to determine the MIC (EUCAST approach). Furthermore, the results were expressed in the percentage of inhibition of growth and can be used to obtain EC_50_ and EC_80_ (24 h), for an EcoAST.

**Assessment of the ecotoxicological approach**

The results of inhibition growth of *E. coli* ATCC 25922, after performing EUCAST methodology, showed that 0.5 mg/L of TRIM is the lowest concentration that inhibited at least 80 % of *E. coli* ATCC 25922 growth (Table S1). These results corroborate the EUCAST database, which reported a MIC value of 0.5 mg/L of TRIM (EUCAST 2023).

According to the EcoAST, results showed that 0.39 mg/L of TRIM inhibits ~ 67.44 % of *E. coli* growth, while 0.78 mg/L of TRIM inhibits ~ 93.74 % of *E. coli* growth. Thus, the concentration that inhibited at least 80 % of *E. coli* growth (MIC) is greater than 0.39 and less than 0.78 mg/L (Table S2). These results showed that despite the adaptations made to the concentrations and volumes tested, the ecotoxicological approach can be implemented, as it allows for obtaining concordant results with the results obtained in the EUCAST methodology (MIC = 0.5 mg/L) (EUCAST 2023). Furthermore, it was also possible to corroborate this agreement by determining the EC_80_ value, which in this case was 0.49 mg/L. In the context of antibiotics, EC_80_ and MIC can be important metrics used to describe antibiotics' effectiveness, but with different purposes. EC_80_ is more related to the antibiotics’ toxicity and the level of response, while MIC is specifically about the minimum concentration required to inhibit bacterial growth.

**Table S1.** Percentage of inhibition growth of *Escherichia coli* ATCC 25922 after exposure to Trimethoprim concentrations (mg/L) according to EUCAST methodology. The MIC value is highlighted in green.

|  | **TRIM concentrations (mg/L)** | | | | | | | | | | | | | |  |  |  |  |  |  |  |  |  |  |  |  |
| --- | --- | --- | --- | --- | --- | --- | --- | --- | --- | --- | --- | --- | --- | --- | --- | --- | --- | --- | --- | --- | --- | --- | --- | --- | --- | --- |
|  | **GC*** | **0.008** | **0.016** | **0.03** | **0.06** | **0.125** | **0.25** | **0.5** | **1** | **2** | **4** | **8** | **16** | **32** |  |  |  |  |  |  |  |  |  |  |  |  |
| **Inhibition of growth (%)** | 0 | 8.40 | 6.97 | 5.81 | -27.37 | 36.79 | 78.44 | 91.85 | 97.36 | 98.48 | 98.96 | 99.48 | 99.04 | 101.48 |  |  |  |  |  |  |  |  |  |  |  |  |
|  | 0 | 11.24 | 1.86 | -1.34 | -5.77 | 54.96 | 77.72 | 95.49 | 98.44 | 98.68 | 98.60 | 99.24 | 99.36 | 100.48 |  |  |  |  |  |  |  |  |  |  |  |  |
|  | 0 | -16.95 | -2.46 | 11.92 | 2.85 | 15.71 | 81.35 | 93.45 | 98.36 | 99.84 | 98.32 | 99.08 | 99.52 | 101.28 |  |  |  |  |  |  |  |  |  |  |  |  |
|  | 0 | -9.76 | -7.29 | -14.99 | 11.04 | 36.83 | 63.43 | 94.17 | 83.43 | 98.68 | 99.00 | 99.36 | 99.08 | 99.80 |  |  |  |  |  |  |  |  |  |  |  |  |
| Average | 0 | 9.82 | 4.41 | 8.86 | 6.95 | 36.08 | 75.23 | 93.74 | 94.40 | 98.92 | 98.72 | 99.29 | 99.25 | 100.76 |  |  |  |  |  |  |  |  |  |  |  |  |
| Values in red were considered outliers and were not considered for the calculated average.  *GC - Growth control. | | | | | | | | | | | | | | |  |  |  |  |  |  |  |  |  |  |  | Values in red were considered outliers and were not considered for the calculated average. The MIC value is marked in green. |

| **Table S2.** Percentage of inhibition growth of *Escherichia coli* ATCC 25922 after exposure to Trimethoprim concentrations (mg/L) according to EcoAST procedure. The MIC value is between the concentrations highlighted in green and orange. | **TRIM concentrations (mg/L)** | | | | | | | | | | | | | | | | | | |
| --- | --- | --- | --- | --- | --- | --- | --- | --- | --- | --- | --- | --- | --- | --- | --- | --- | --- | --- | --- |
|  | **GC*** | **0.0015** | **0.003** | **0.006** | **0.01** | **0.02** | **0.05** | **0.1** | **0.195** | **0.39** | **0.78** | **1.56** | **3.125** | **6.25** | **12.5** | **25** | **50** | **100** | **200** |
| **Inhibition of growth (%)** | 0 | 4.36 | 5.44 | 5.92 | 5.53 | 3.05 | 4.97 | 3.27 | 17.38 | 80.57 | 96.73 | 71.78 | 100.3 | 99.56 | 99.39 | 100.2 | 100.0 | 100.1 | 102.6 |
|  | 0 | -3.09 | 2.66 | -0.57 | 3.79 | 2.66 | -0.30 | 1.87 | 39.55 | 75.91 | 98.65 | 100.5 | 101.1 | 100.9 | 95.08 | 100.9 | 99.78 | 99.52 | 103.1 |
|  | 0 | 4.88 | 4.49 | 2.48 | 4.01 | 2.26 | 2.66 | -6.27 | 8.58 | 30.40 | 79.14 | 83.06 | 101.1 | 89.50 | 100.8 | 101 | 100.6 | 100.1 | 102.8 |
|  | 0 | -17.60 | -6.23 | -13.37 | -14.68 | -13.76 | -23.56 | -44.34 | 31.32 | 82.88 | 97.47 | 99.91 | 99.61 | 99.70 | 91.29 | 100.9 | 99.43 | 98.95 | 102.7 |
| Average | 0 | 4.62 | 4.20 | 4.20 | 4.44 | 2.66 | 3.81 | 2.57 | 24.21 | 67.44 | 93.00 | 88.81 | 100.5 | 97.42 | 96.64 | 100.8 | 100.0 | 99.67 | 102.8 |
| Values in red were considered outliers and were not considered for the calculated average.  *GC - Growth control. | | | | | | | | | | | | | | | | | | | |

**References**

EUCAST, 2023. The European Committee on Antimicrobial Susceptibility Testing. Routine and extended internal quality control for MIC determination and disk diffusion as recommended by EUCAST.

EUCAST, 2022. EUCAST reading guide for broth microdilution. Version 4.0.

Sigma Aldrich, 2022. Trimethoprim - Sigma Aldrich product information.
